# Rapid emergence of independent “chromosomal lineages” in silvered-leaf monkey triggered by Y/autosome translocation

**DOI:** 10.1038/s41598-018-21509-4

**Published:** 2018-02-19

**Authors:** Oronzo Capozzi, Roscoe Stanyon, Nicoletta Archidiacono, Takafumi Ishida, Svetlana A. Romanenko, Mariano Rocchi

**Affiliations:** 1 Department of Biology, University of Bari, Bari, Italy; 2 Department of Biology, University of Florence, 50122 Florence, Italy; 3 Department of Biological Sciences, Human Biology & Genetics, Graduate School of Science, University of Tokyo, 113-0033 Tokyo, Japan; 4 Institute of Molecular and Cellular Biology SB RAS, 630090 Novosibirsk, Russia; 5 Novosibirsk State University, 630090 Novosibirsk, Russia

## Abstract

Sex/autosome translocations are rare events. The only known example in catarrhines is in the silvered-leaf monkey. Here the Y chromosome was reciprocally translocated with chromosome 1. The rearrangement produced an X_1_X_2_Y_1_Y_2_ sex chromosome system. At least three chromosomal variants of the intact chromosome 1 are known to exist. We characterized in high resolution the translocation products (Y_1_ and Y_2_) and the polymorphic forms of the intact chromosome 1 with a panel of more than 150 human BAC clones. We showed that the translocation products were extremely rearranged, in contrast to the high level of marker order conservation of the other silvered-leaf monkey chromosomes. Surprisingly, each translocation product appeared to form independent “chromosome lineages”; each having a myriad of distinct rearrangements. We reconstructed the evolutionary history of the translocation products by comparing the homologous chromosomes of two other colobine species: the African mantled guereza and the Indian langur. The results showed a massive reuse of breakpoints: only 12, out of the 40 breaks occurred in domains never reused in other rearrangements, while, strikingly, some domains were used up to four times. Such frequent breakpoint reuse if proved to be a general phenomenon has profound implications for mechanisms of chromosome evolution.

## Introduction

The X and Y chromosomes are rarely involved in translocations due to their role in sex determination. Evidently, sex chromosome rearrangements are characterized by lowered fitness. Apparently, when sex chromosome are involved in translocations they are rapidly eliminated by selection. In placental mammals, comparative studies have repeatedly confirmed that the X-chromosome is the most highly conserved chromosome with respect both to gene content and marker order^1,2^. In contrast, the Y chromosome is often morphologically distinct even in closely related mammalian species, due to a highly variable and rapidly evolving content of repeat DNA^3^. This variability is often notable even within the same species. For example, satellite DNA in humans can form variable blocks up to several Mb in size, and the size of the Y-chromosome follows a normal distribution in the population^4^.

In catarrhine primates (Old World monkeys, apes and humans) only one species is known to have a sex/autosome translocation. This very rare exception involves chromosome Y and chromosome 1 in the silvered-leaf monkey, *Trachypithecus cristatus* (TCR, formerly *Presbytis cristatus*), a Southeast Asian colobine monkey. The peculiar t(1;Y) translocation found in TCR was first reported by Dutrillaux *et al*.^5^ using R-banding. Later, Bigoni *et al*.^6^ investigated this translocation using molecular cytogenetics techniques. They mapped the homology between all human chromosomes and those of the silvered-leaf monkey with chromosome paints and showed that the Y chromosome was reciprocally translocated with TCR chromosome 1 (TCR1; entirely homologous to human chromosome 5, HSA5). This translocation produced a X_1_X_2_Y_1_Y_2_ sex chromosome system (sex chromosomes were considered as all the chromosomes which were unpaired in the male). X_1_ equals the normal X chromosome, X_2_ equals TCR1; Y_1_ and Y_2_ equal the reciprocal translocation products t(1;Y). They examined ten TCR individuals in all, five females and five males, and found that the untranslocated TCR1 was present in 3 polymorphic forms, TCR1a/b/c. Ponsà *et al*.^7^ had previously reported that this species had two variants of chromosome 1. The t(1;Y) translocation was recently studied by Xiaobo *et al*.^8^ using the multicolor banding consisting of pools of microdissected painting probes and five BAC clones from HSA5.

Our previous investigations in primates revealed that chromosomes homologous to the TCR1 are normally highly conserved. For example, the human, orangutan and macaque homologs have an identical marker order^9^, inherited unchanged from the corresponding chromosome of the Common Catarrhine Ancestor (CCA4)^10^. The FISH experiments performed in the present study, using a panel of more than 150 human BAC clones, showed that Y_1_, Y_2_, and TCR1 chromosomes, contrary to the rest of the highly conserved TCR complement, were inundated by a series of inversions. Surprisingly, most of the rearrangements found in TCR1, Y_1_, and Y_2_ chromosomes were independent. Each translocation product apparently formed separate “chromosomal lineages”. Unexpectedly, we also found that breakpoints were frequently reused. In 28 out of the 40 breakpoints, one or both margins were reused up to four times in other rearrangements.

## Results

**Figure 1:**
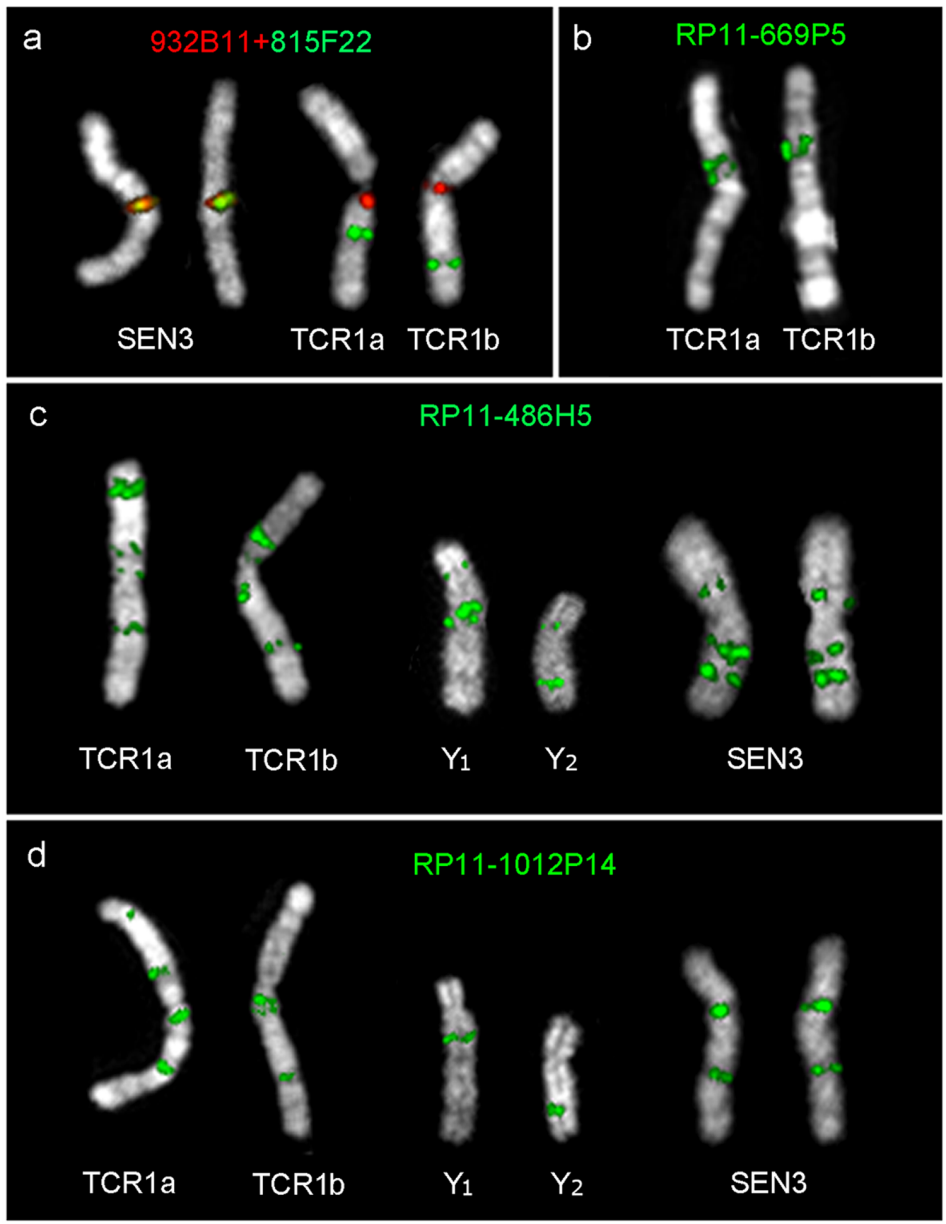
Examples of FISH experiments. All the BAC clones used in the FISH experiments belong to the RP11 BAC library. (**a**) The FISH signals of the two BACs RP11-932B11 (chr5:53,947,332-54,140,932) and RP11-815F22 (chr5:54,253,088-54,457,288) overlap in the human sequence and in SEN3 (left), but are split apart by the upper breakpoint (green arrow in Fig. 2a) of the inversion involving blocks #5, #6, and #12, separating blocks #4 and #5 in the trajectory LCA → TCR1a. The two BACs RP11-932B11 and RP11-815F22, therefore, delimit the margins facing the breakpoint of the blocks #4 and #5, respectively. The apparent different position of BAC RP11-815F22 in TCR1b with respect to TCR1a is the result of subsequent inversions occurred in the line to TCR1b. (**b**, **c** and **d**) Examples of duplicated signals produced by BACs belonging to the duplicated segments mapping, in humans, at 34 Mb, 168 Mb, and 174 Mb respectively, as reported in Table 1.

**Figure 2:**
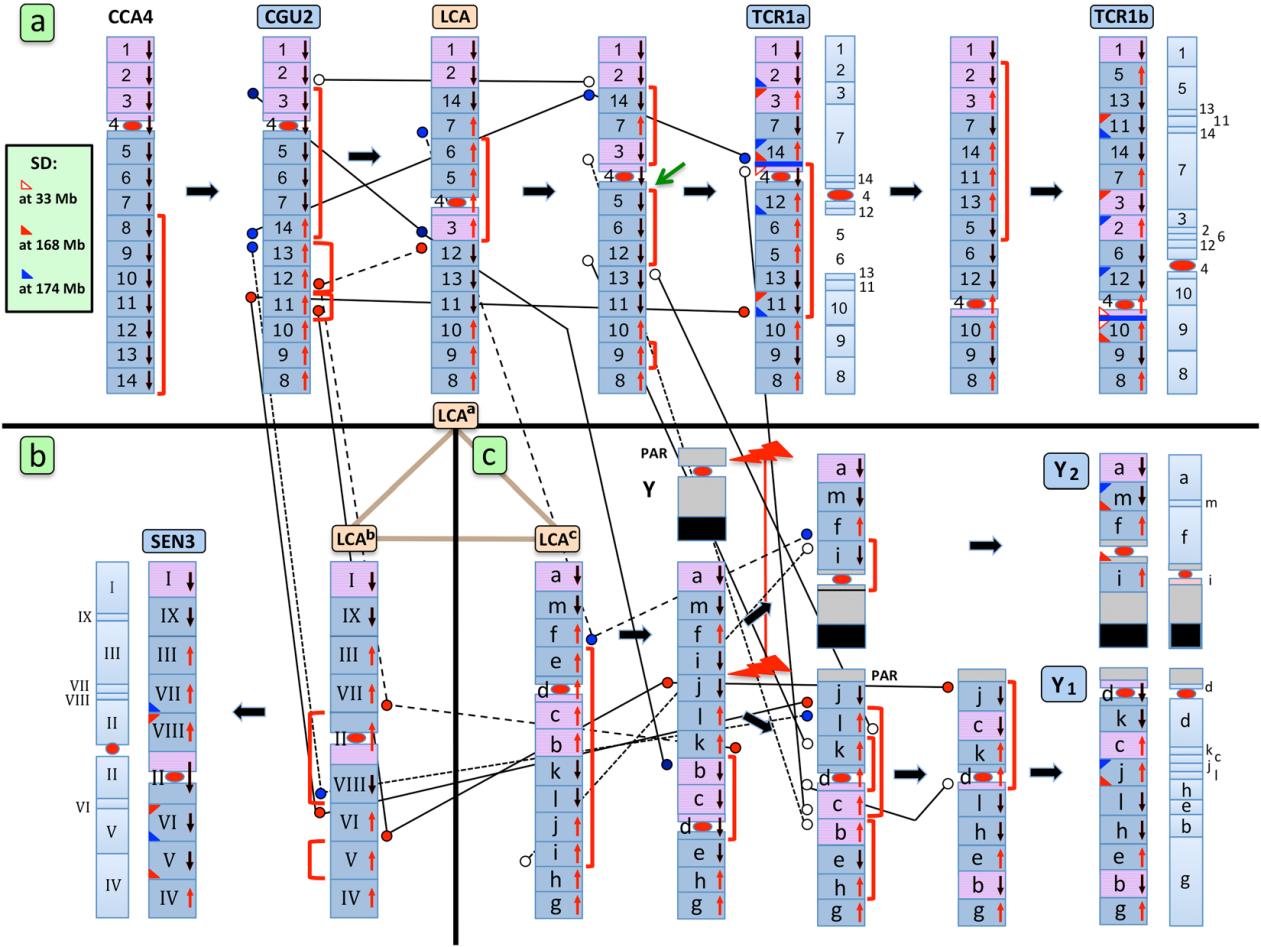
Evolutionary history of CGU2, TCR1a/b, Y_1_, Y_2_, and SEN3 chromosomes. The Figure shows the different synteny blocks arrangement of the chromosomes under study, generated by rearrangements occurred since their catarrhini common ancestor (CCA4). Each synteny block was drawn, for clarity, as one unit, regardless of its length in nucleotides. Some blocks were very small (few Mb) and would be difficult to illustrate in the figure otherwise. The ideogram on the right of TCR1a, TCR1b, Y_1_, and Y_2_, and on the left of SEN3 roughly represents the relative size of blocks (for details see Supplemental File 1). The same LCA intermediate was shown three times, at beginning of each of the three evolutionary lines (TCR1a/b, Y_1_, Y_2_, and SEN3). The three LCA ideograms appear different in (**a**), (**b**), and (**c**) because LCA^b^ and LCA^c^ were drawn to reflect the position, in LCA, of blocks composing SEN3, Y_1_, and Y_2_, respectively. The sequence orientation of each block with respect to CCA4 is indicated by an arrow on the right of the block. Down-pointing black arrows and up-pointing red arrows indicate concordant or reverse sequence orientation with respect to the human sequence, respectively. Inversions are represented by a red parenthesis on the right or left of the ideograms. TCR1b and Y_2_ arms are represented upside down to better illustrate the flow of rearrangements and block orientation. Dots external to blocks (on left or right) and close to the upper or lower margin, indicate that the margin was reused two times (white dots), three times (blue dots), or four times (red dots). The lines connecting dots (i.e. same margins) were arbitrarily drawn in different shapes just to make them more easily distinguishable each other. Segmental duplication (SD) were annotated as small triangles adjacent to the margin where the FISH signal was detected, as indicated in the box in the upper left side. Multiple FISH signals generated by BACs mapping at 33–34, 168, and 174 Mb are represented by empty triangles, red triangles, and blue triangles, respectively. Details on these BACs are reported in Table 1. The thick blue segment between blocks 4/14 and 4/10 present in TCR1a and TCR1b respectively, indicates the localization of the extra-band lit up by the total TCR genomic DNA (see Fig. 4). The green arrow in Fig. 2a points to a breakpoint illustrated in Fig. 1a. For details see text.

TCR1 and the t(1;Y) reciprocally translocated chromosomes Y_1_ and Y_2_ were investigated by FISH using a panel of up to 70 human end-sequenced BAC clones evenly distributed along human chromosomes 5 and Y, chosen from the UCSC BAC track (https://genome.ucsc.edu), hg19 release, to which all the present sequence data are referred. Metaphase preparations were obtained from lymphoblastoid cell lines from one male and one female TCR monkey. BACs were cohybridized, two or three at a time, in search of synteny disruptions with respect to CCA4, which is identical, as mentioned, to HSA5^10^. Hereafter, CCA4 and HSA5, as far as marker order is concerned, will be used synonymously. When a synteny disruption was detected, we then performed reiterative FISH experiments to precisely characterize each breakpoint. We mapped each breakpoint within a BAC, which yielded a split signal, or between overlapping BACs mapping to each side of the breakpoint, as in the example in Fig. 1a. The FISH experiments showed that the two TCR1 homologs of the female differed. This result was not unexpected because previous publications reported that this chromosome was polymorphic in the species (see above). The female we studied had forms classified from banding as TCR1a and TCR1b. The single intact chromosome 1 of the male was TRC1a. The synteny blocks organization of these two TCR1 forms and of Y_1_-Y_2_ translocated chromosomes are reported in detail in Supplemental File 1 and, graphically, in Fig. 2.

One goal of this study was to track the series of rearrangements that comprise the evolutionary history of TCR1 variants and the t(1;Y) translocation products from the ancestral CCA4 chromosome. To better track the flow of rearrangements we hybridized an appropriate subset of the BAC clones to metaphase chromosomes of two additional colobine species available to us: *Colobus guereza* (CGU; mantled guereza) and *Semnopithecus entellus* (SEN; Indian langur). CGU is an African colobine, and is an appropriate outgroup to TCR and SEN (Asian colobines)^11,12^. In CGU and SEN the chromosomes corresponding to TCR1 are CGU2 and SEN3, respectively^13^. Synteny disruptions were refined with reiterative BAC-FISH experiments as above. The detailed results are reported in Supplemental File 1. We then compared the synteny block organization of TCR1a/b, Y_1_, Y_2_, CGU2, and SEN3 chromosomes in order to reconstruct a comprehensive evolutionary history of these chromosomes. The reconstruction was aided by the use of the GRIMM software^14^, available online at http://grimm.ucsd.edu/GRIMM.

### Evolutionary history of CGU2, TCR1a/b, Y_1_, Y_2_, and SEN3

The hypothesized evolutionary history of these chromosomes is graphically summarized in Fig. 2. CCA4 was used as the starting point. The short arm is in striped pink. A single paracentric inversion in the long arm of CCA4 generated a chromosome corresponding to the present day CGU2. Three inversions of this form produced an intermediate chromosome that constituted the Last Common Ancestral form of SEN3, TCR1a/b, Y_1_, and Y_2_ (LCA in Fig. 2). Four inversions in this form were necessary to derive the synteny arrangement of TCR1a and two additional inversions gave origin to TCR1b. The 14 synteny blocks found on TCR1b were numbered according to their original position on CCA4/HSA5. Inversions are occasionally reported as apparently simultaneous (present on the same intermediate form). This occurs when two or more consecutive inversions did not overlap and/or when an inversion was nested inside a larger inversion. In these cases, the temporal sequence could not be resolved and they were represented as simultaneous.

**Figure 3:**
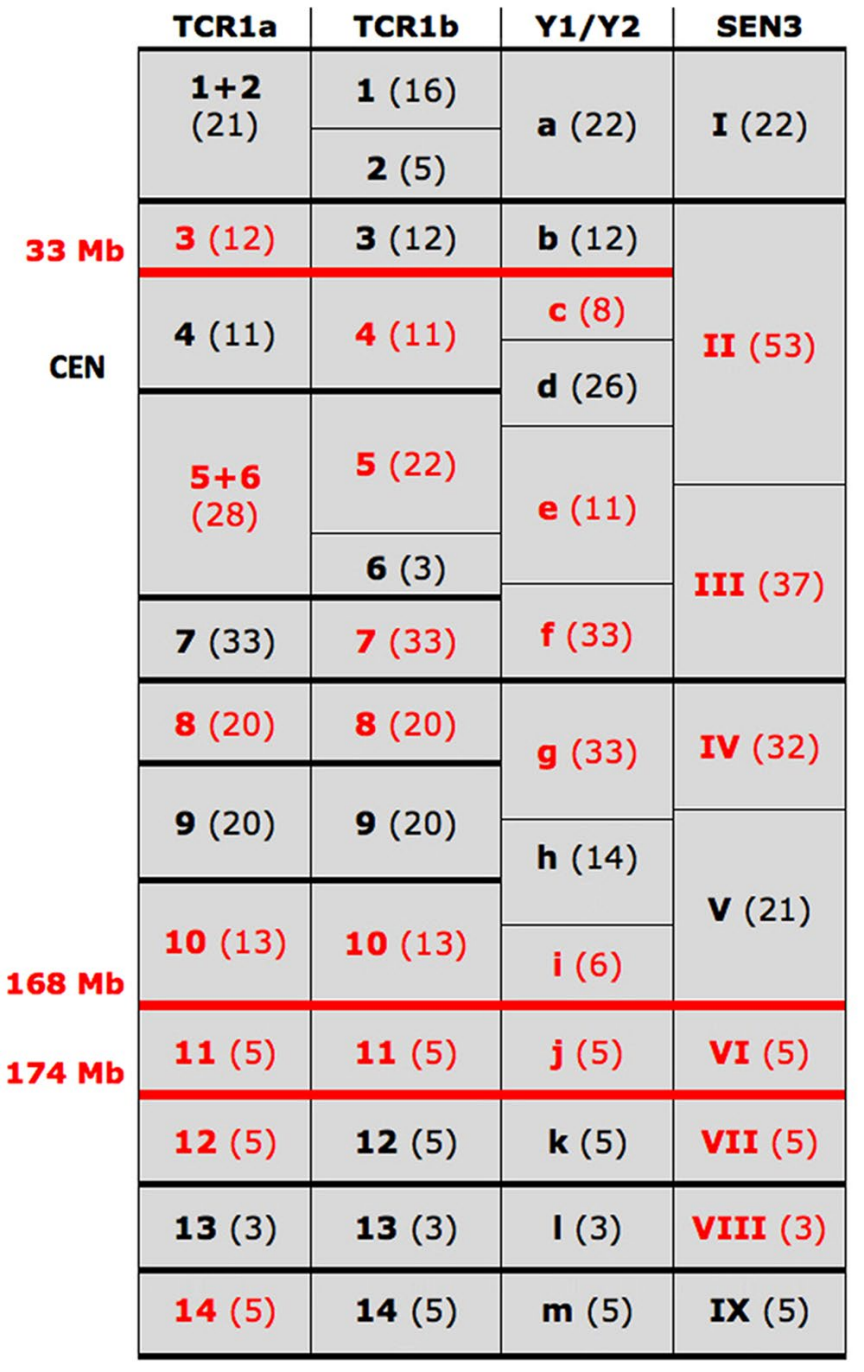
Margins shared among synteny blocks. This Figure simplifies the data of block margins present in columns J/K/L/M of the HSA5 sheet of Supplemental File 1. It shows the reciprocal relationship of the different synteny blocks, for an easy identification of shared margins. Thick segments indicate shared margins. Actual blocks sizes (Mb) are annotated in parenthesis. The orientation is indicated by color: black for forward and red for reverse sequence orientation.

Figure 2b and c report the changes necessary to derive SEN3, Y_1_, and Y_2_ from LCA, respectively. We used different block nomenclatures for these two trajectories: Roman numbers for the LCA to SEN3 and lower letters for LCA to Y_1_ and Y_2_. The different nomenclatures were necessary to avoid confusion among blocks because block size and position in TCR1a/b, Y_1_, Y_2_, and SEN3 vary according to their different evolutionary history (different rearrangements). As a consequence, the three LCA copies reported in Fig. 2a–c (LCA^a^, LCA^b^, LCA^c^) although identical as far as marker order is concerned, appear different because LCA^b^ and LCA^c^ were considered as the starting points of the two trajectories LCA → SEN3 and LCA → Y_1_–Y_2_, respectively. Detailed data on the size and extension of the different synteny blocks can be obtained from Supplemental File 1. Columns J/K/L/M of the first sheet (HSA5) report the relationships of all blocks in the human sequence, allowing the identification of margins shared by blocks present in the chromosomes TCR1a, TCR1b, Y_1_, Y_2_, and SEN3. A simplified view is reported in Fig. 3, from which shared margins can be pinpointed at a glance, thus allowing a more easily analysis of margins’ reuse in the rearrangements (see below).

As mentioned, not all the inversions could be chronologically ordered with certainty. However, we temporally positioned the t(1;Y) translocation event (lightning bolts in Fig. 2c) at an initial stage, just after the inversion that generated a form composed of juxtaposed Y1 and Y2 chromosomes, from which the translocation can immediately follow. This chronology was dictated by the unprecedented finding that most of the rearrangements that occurred in Y_1_ and Y_2_ with respect to TCR1a/b were Y_1_ and Y_2_ or TCR1a/b-specific, as if these chromosomes did not share their recent evolutionary history. We reasoned that an early occurrence of the translocation could have created a meiotic barrier fully accounting for the independent evolution of the two “chromosomal lineages” we have documented.

It was not a simple task to define the organization of the original Y chromosome on Y_1_ and Y_2_. Y chromosomes are mainly composed of duplicated and rapidly diverging repeat sequences and, as a consequence, relatively few human BAC Y clones yielded satisfactory results on TCR. The Pseudo Autosomal Region (PAR) in this respect is an exception because it engages in crossing over the X chromosome. Indeed, the three BACs mapping to the PAR region (see Supplemental File 1) yielded good results and allowed us map the PAR to Y1 (Fig. 2).

**Figure 4:**
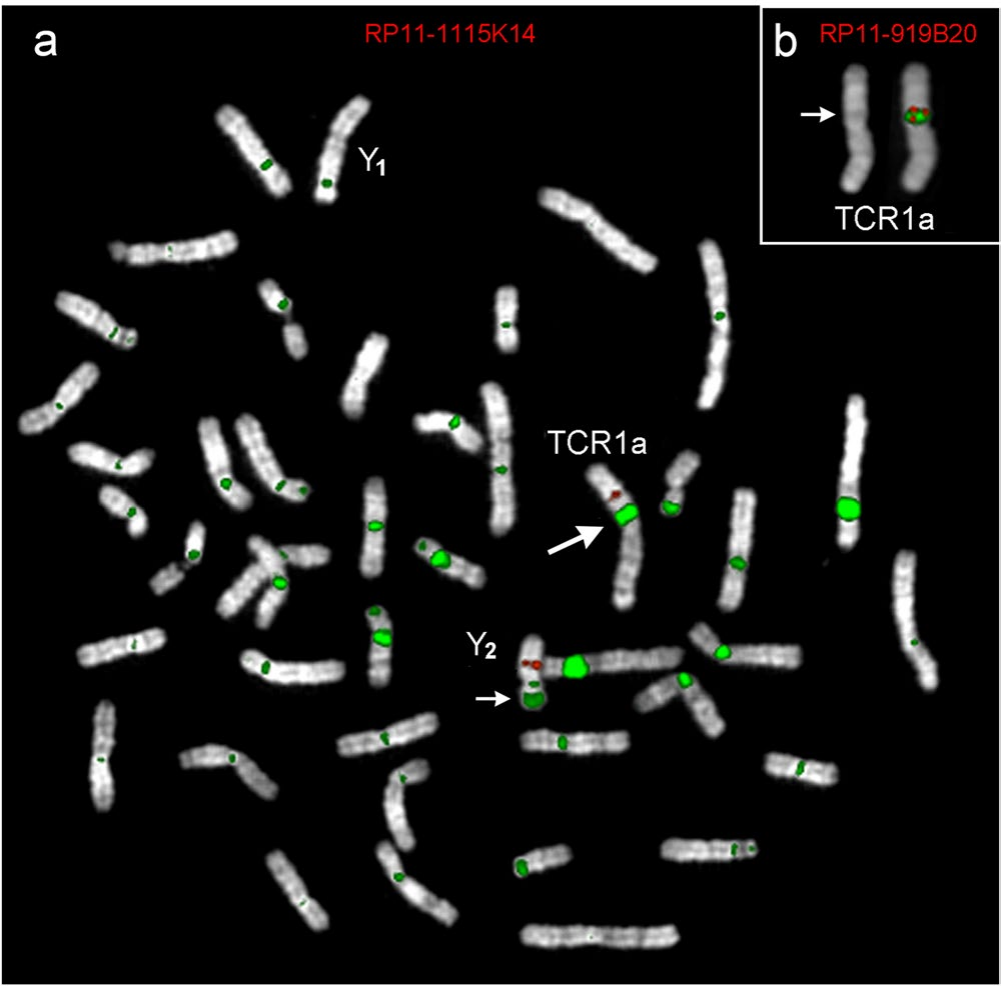
FISH of total TCR genomic DNA on male TCR metaphase. (**a**) Total genomic DNA was used as probe in high stringency FISH experiments on male TCR metaphase (signals in green). The red signal is due to BAC RP11-1115K14 used as a marker for Y_2_ identification. The BAC maps, in humans, to chr5:96,675,964-96,821,846, inside block #7 of TCR1 and block “f” of Y_2_ (see Fig. 3). The small arrow points to the heterochromatic block of the distal part of chromosome Y present in Y_2_ (see Fig. 2c). The large arrow points to the extra-band on TCR1, absent in Y_1_ and Y_2_. Note the large size variations among centromeres of different chromosomes. (**b**) A partial metaphase showing a cohybridization experiment of total male TCR genomic DNA (green) and BAC RP11-919B20 (red) on the male metaphase, which yielded duplicated signals surrounding the extra-band. The BAC belong to the duplicated domain at 33–34 Mb (see Table 1). The DAPI banding alone is shown on the left to show that the extra-band appear pale as does the centromeric satellite DNA.

We then hybridized total male TCR genomic DNA, at high stringency, to map the major blocks of satellite DNA present in this species. The result is shown in Fig. 4. All centromeres had FISH signals, with large size variation among chromosomes. In addition to centromeric domains, the experiment revealed two non-centromeric FISH signals. A large signal was found on the telomere of the Y_2_ short arm (small arrow), corresponding, very likely, to the distal long arm of the untranslocated chromosome Y (in black in the diagram of Fig. 2c). A second and totally unexpected non-centromeric signal was detected on TCR1a and TCR1b (big arrow in the metaphase of Fig. 4, from a male individual). It mapped at the boundary between blocks 4/14 in TCR1a and between blocks 4/10 in TCR1b (in blue in Fig. 2). Even more surprisingly, this extra heterochromatin band (hereafter extra-band) was absent in the corresponding region of Y_2_ (for details see the legend to Fig. 4).

### Segmental duplications

Some BACs which yielded single signals in human, macaque, and CGU produced, instead, multiple signals in TCR and SEN indicating that segmental duplications were clearly active in the Asian colobine homologs to HSA5/CCA4. Sequences corresponding to BACs that were split by a breakpoint were obviously not considered as duplicated. Table 1 list the BAC clones yielding duplicated signals, located in domains at 33–34, 168, and 174 Mb in the human hg19 release. FISH examples are reported in Fig. 1b–d. The duplicated FISH signals are annotated in Fig. 2 as small triangles positioned on the left of the block, facing the upper or lower margin according to the FISH data. It should be noted that SDs were mainly found at boundaries of blocks #11, #12, #13, and #14 of TCR1a/b, and their corresponding segments in Y_1_-Y_2_ and SEN (see Fig. 3). These blocks are relatively small. Therefore, the precise SD localization with respect to the block margins could not be determined with absolute certainty.

**Table 1:** BAC clones producing multiple signals.

Domain	BAC	Band	hg19	TCR1a	TCR1b	Y1	Y2	SEN3
33/34 Mb	RP11-845K11		chr5:33,153,052-33,331,631	1p	1qcen	*Y1q*	*y2q*	3p
	RP11-586D11		chr5:33,298,621-33,490,405	*1pdup*	*1qcen* + *1p*	*Y1q dup*	*y2q*	3p
	RP11-669P5		chr5:33,698,253-33,885,821	*1pdup*	*1pdup*	*Y1q*	*y2cen*	3p
	RP11-94E6	5p13.3	chr5:33,701,513-33,890,257	*1pdup*	*1pdup*	*Y1q*	*y2cen*	3p
	RP11-664P14		chr5:33,884,719-34,053,037	*1pdup*	*1pdup*	*Y1q*	*y2cen*	3p
	RP11-919B20		chr5:34,402,851-34,576,348	*1pdup*	*1pdup*	Y1q		3p
	RP11-55N11		chr5:34,432,387-34,620,014	*1pdup*	*1pdup*	y1q		3p
168 Mb	RP11-593P12		chr5:168,598,103-168,759,041	1q	1p		Y2pcen	*3q split*
	RP11-626A11		chr5:168,731,124-168,906,232	1q	1p		Y2pcen	*3q split*
	RP11-805C8	5q35.1	chr5:168,877,515-169,043,723	*1q* + *1p*	*1p* + *1q*	*Y1q*	*Y2pcen* + *Y2q*	*3 signals*
	RP11-486H5		chr5:168,976,857-169,159,786	*4 signals*	*4 signals*	*Y1pcen* + *Y1q*	*Y2pcen* + *Y2q*	*3 signals*
	RP11-927B5		chr5:169,155,125-169,347,618	*1p* + *1q*	*1p* + *1q*	Y1q		3q
174 Mb	RP11-768K12		chr5:174,234,491-174,402,010	*dup*	*dup*	*Y1q*	*Y2q*	*3q* + *3p*
	RP11-1012P14	5q35.2	chr5:174,303,447-174,508,861	*4 signals*	*1qcen* + *1q* + *1p*	*Y1q*	*Y2q*	*3p* + *3q*

Chromosomes where multiple signals were found are in Italics.

We then proceeded to investigate further the composition of the extra-band to test the hypothesis that it could be due to an enlarged domain of segmental duplications. We cohybridized BACs from the three SD domains with total genomic DNA of the male individual. No overlap between SD signals and the extra-band domains was found. Figure 4b, for example, clearly shows that the duplicated BAC RP11-919B20 (domain at 33–34 Mb) distinctly flanks the extra-band. This Figure also shows that the extra-band appears pale in DAPI staining, similar, in intensity, to the centromeric heterochromatin domains. We could conclude, therefore, that the extra-band was probably form by sequences of repeat DNA.

### Breakpoint reuse

The 40 breakpoints we documented in the three studied species generated 27 distinct blocks, as shown in Supplemental File 1 and in Fig. 3. Notably, in 28 out of the 40 breakpoints, one or both margins were reused in other rearrangements, and only two inversions of the 20 rearrangements (19 inversions and one translocation) had both breakpoints falling in domains that were used just once. The reused margins were annotated with dots positioned on left or right of the block, with lines connecting corresponding margins, indicating the reuse. Dots of different color distinguish margins that were reused two (white dots), three (blue), or four times (red). In detail: 7 margins were used two times, 4 margins three times, and 3 margins four times.

## Discussion

By performing FISH experiments using a large panel of human BAC clones we were able to characterize in detail TCR1a and TCR1b, the two forms present in the two individuals available to us, and the Y_1_ and Y_2_ translocation products. This detailed characterization can be used, in future studies, as a reference in defining additional TCR1 forms that may be present in the silvered-leaf monkey. Furthermore, having analyzed a single TCR male individual, we cannot discard with certainty the possibility that also Y_1_ and Y_2_ could be polymorphic in the population. Cytogenetic data available in the literature, however, do not support this hypothesis.

We defined the evolutionary history of TCR1a, TCR1b, Y_1_, and Y_2_ from the common catarrhine ancestor. The results are summarized in Fig. 2 and in Supplemental File 1. The two TCR1 chromosomes and the Y_1_ and Y_2_ translocation products had an astonishing number of rearrangements compared to other TCR chromosomes. The analysis also revealed, surprisingly, that most of the rearrangements found in Y_1_ and Y_2_ were independent from those that produced TCR1a and TCR1b.

### Evolutionary history of TCR1, Y1-Y2, CGU2, and SEN3

The BAC-FISH experiments were extended to an additional Asian colobine, *Semnopithecus entellus* (SEN), and an African colobine *Colobus guereza* (CGU), which provided data to reconstruct the evolutionary history of TCR1. The ancestor of all catarrhines had a homolog (CCA4) perfectly conserved in humans, orangutans, and macaques. Thus, this chromosome remained unchanged in these species for more than 25 million years. Colobines diverged from other catarrhine monkeys about 18 mya and now comprise more than 60 species in multiple genera distributed from Africa to Asia^11^. In colobines this chromosome was subject to multiple rearrangements. The first rearrangement was a paracentric inversion. The resulting chromosome is found today in the African *Colobus guereza* (CGU2) (Fig. 2a). This synapomorphic inversion phylogenetically links all colobine monkeys^10^, therefore it occurred in the common African/Asian colobine ancestor. Two paracentric and a pericentric inversions then occurred in the CGU2 chromosomal form, generating the LCA form. We carefully considered if the LCA form was actually the last common form with respect to TCR1a, TCR1b, Y_1_, and Y_2_. We examined, exploiting the GRIMM software, the potential different forms generated by the inversions when introduced in different chronologies, but no alternatives were found. We also considered the possibility that Y_1_ and Y_2_ could be more related to the TCR1c than to TCR1a and b. After a careful examination of the banding pattern of TCR1c, as reported by Bigoni *et al*.^6^ we excluded this possibility. The BAC-FISH analysis of additional colobine species might eventually clarify if this LCA form is still present in one or more extant colobine species.

Four additional inversions generated TCR1a and two further inversions resulted in TCR1b. In all, the TCR1b was dissected in 14 different synteny blocks with respect to CCA4. Some of the inversions reported in the Fig. 2 could not be resolved temporally (see above), but the chronological order of the inversions does not affect either number of breakpoints or synteny blocks.

Figure 2c reports the hypothesized series of rearrangements that remodulated LCA into Y_1_ and Y_2_. GRIMM analysis provided additional alternative temporal scenarios in which the translocation event could be postulated even as the last event. However, we regarded an early t(1;Y) translocation as a necessary and sufficient meiotic barrier able to explain the independent evolution of TCR1, Y_1_, and Y_2_. The first inversion reported in the path LCA → Y_1_ and Y_2_ was introduced first because it was necessary to generate a chromosomal form from which Y_1_ and Y_2_ can be immediately derived via the reciprocal translocation with chromosome Y. In this context the absence of the intriguing extra-band detected on TCR1a and TCR1b, but absent on Y_2_ indicates that it originated *de novo* after the translocation event. This finding is further evidence in support of the independent evolution of TCR1 from Y_1_ and Y_2_. We hypothesize that the extra-band is composed of satellite DNA and its molecular characterization might help shed light on how heterochromatic blocks can be rapidly formed.

Data from other Asian colobines and, in particular, species from the genus *Trachypithecus* might well be relevant to further and most precisely dissecting the evolutionary history of TCR1. Unfortunately, we did not have, at this time, further Asian colobine cell lines available for study. Therefore, we carefully consulted the literature to analyze all the available karyotypes of species from this genus. The translocation was found absent in the *Trachypithecus obscurus*^15^, but present, unreported, in *Trachypithecus germaini germaini*^16^. According to Springer *et al*.^12^ *T. cristatus, T. germaini*, and *T. auratus* form a clade which diverged, about 2 mya, from the sister clade *T. obscurus*, *T. barbei*, and *T. phayrei*. Then, about 0,8 mya, *T. auratus* diverged from *T. cristatus* and *T. germaini*. The latter two species, after a very short period of common descent, separated from each other. In summary, it can be concluded that the t(1;Y) translocation occurred between 2,0 and 0,8 mya. This phylogenetic scheme provides a time interval in which the marker order of Y_1_, Y_2_, and TCR1 diverged. In order to better date the translocation it would be useful to know if *T. auratus* also has the t(1;Y) translocation. If not, then the event could be dated at about 0,8 mya and, consequently, the Y_1_, Y_2_, and TCR1 could be hypothesized to have occurred in a relatively short period of time. Extremely rapid chromosomal divergence is not rare and good examples, as mentioned, are gibbons and equids^17,18^.

The portion of chromosome Y present in Y_2_ appears relatively large with respect to the average chromosome Y size in primates. However, this finding is on line with the large chromosome Y present in *T. obscurus*^15^, a species phylogenetically close to *T. cristatus*^12^.

### TCR1 accelerated evolution

We have mentioned above two examples of rapid karyotype evolution (gibbons and equids). Usually, however, the rapid evolution involves all or almost all chromosomes. In our case just a single chromosome was involved. The assertion that no other TCR chromosomes were affected by deep restructuring, as opposite to TCR1, is not based just on banding pattern analysis or on chromosome painting studies, that are not very efficient in detecting inversions, but relies on detailed BAC-FISH analysis we already reported in publications on the evolution of primates’ chromosome 3^19^, 6^20^, 10^21^, 11^22^, 13^23^, 18^24^, and 20^25^. Note that in these papers the TCR is reported as *Presbytis cristatus*, PCR. In addition, we have unpublished BAC-FISH data on chromosomes 2, 4, 7, 8, 12, 14, 15, 16, and 19 also supporting our conclusion.

### Segmental duplications, chromosomal rearrangements and breakpoint reuse

The SDs detected by the three clusters of BAC clones reported in Table 1 never yielded signals on chromosomes other than TCR1, further supporting the unique features of this chromosome. In addition, we detected SD FISH signals in 7 out of 13 breaks. This observation strongly suggests a link between SDs and rearrangements, as already reported in the literature^26^. They also might well be relevant to explain the breakpoint reuse. In Fig. 2 the white, blue, or red dots external to blocks (on left or right), close to the upper or lower margin, indicate that that margin was reused two times, three times, or four times, respectively. The reuse, outlined by connecting lines (see Fig. 2), was extensive: out of the 40 breakpoints we detected, one or both margins were reused in 28 breakpoints, and only one inversions had both breakpoints encompassing never reused chromosomal domains. The relationship SD-breakpoints could well be linked in a feedback mechanism. The inversions could amplify the extent of a SD, which, in turn, could increase the chance of a further involvement of the SD in rearrangements. A fully, high coverage sequencing of TCR is therefore welcomed to elucidate all the hypotheses we mentioned in the present paper, with particular reference to the relationship among the SDs which is difficult to establish with molecular cytogenetics alone. In this context is also worth noting that the synteny block arrangement and orientation we provide in the present paper would be of help in the correct sequencing assembly of the complex chromosomes TCR1, Y_1_, andY_2_.

As mentioned, TCR is the only known catarrhine species showing a Y/autosome translocation. Other cases of Y/autosome translocations have been reported in the New World Monkey genera *Aotus*^27^, *Callimico*^28^, and *Alouatta*^29,30^. However, these cases have at best only been studied with chromosome painting and others only with chromosome banding. We are planning a future research project to study some of these sex chromosome systems using BAC-FISH.

## Methods

### FISH

Metaphase preparations were obtained from lymphoblastoid cell lines of *Trachypithecus cristatus* (one male and one female), and *Semnopithecus entellus* (one female) which were all transformed by Takafumi Ishida (University of Tokyo, Japan); the male *Colobus guereza* was that previously reported^31^. The preparations were obtained using standard methods. No intervention on live animals was performed.

BAC clones were from the RP11 human library^32^. Extraction of total DNA from BACs was performed according to standard methods. Chromosome preparations were hybridized *in situ* with probes directly labeled with Cy3-dCTP, FluorX-dCTP, DEAC, or Cy5-dCTP by nick-translation. Briefly, 300 ng of labeled probe (total BAC DNA) was used for the FISH experiments; hybridization was performed at 37 °C in 10 mL of hybridization buffer containing 2xSSC, 50% (v/v) formamide, 10% (w/v) dextran sulfate, 5 mg of Cot1 DNA (Roche), and 3 mg of sonicated salmon sperm DNA. Post-hybridization washes of FISH experiments were performed at lower stringency 37 °C in 2xSSC-50% formamide (three times), followed by three washes at 42 °C in 2xSSC (three times). Chromosome identification was obtained by simultaneous DAPI banding.

Digital images were obtained using a Leica DMRXA2 epifluorescence microscope equipped with a cooled CCD camera (Princeton Instruments). FluorX, Cy3, DEAC, Cy5, and DAPI fluorescence signals, detected with specific filters, were recorded separately as grayscale images. Pseudocoloring and merging of images were performed using Adobe Photoshop^TM^ software.

All methods were carried out in accordance with relevant guidelines and regulations.

## Electronic supplementary material

Supplemental File 1
